# Genome Sequences of Mycobacteriophages Findley, Hurricane, and TBond007

**DOI:** 10.1128/genomeA.01123-17

**Published:** 2017-11-09

**Authors:** Sanghamitra Saha, Wenbo Yan, Jacqueline M. Washington, William B. Davis, W. Brad Barbazuk, Rosa Medellin, Aissa Mendez, Jacqueline R. Ramos, Rachna Sadana, Miranda Edmonds, Guanqiao Feng, Maria Ilyas, Joshua Kaiser, Stephen J. Monroe, Thinh V. Pham, Steven G. Cresawn, Rebecca A. Garlena, Ty H. Stoner, Daniel A. Russell, Welkin H. Pope, Deborah Jacobs-Sera, Graham F. Hatfull

**Affiliations:** a Department of Biology, University of Houston-Downtown, Houston, Texas, USA; b Department of Biology and Chemistry, Nyack College, Nyack, New York, USA; c School of Molecular Biosciences, Washington State University, Pullman, Washington, USA; d Department of Biology and Genetics Group, University of Florida, Gainesville, Florida, USA; e Department of Biology, James Madison University, Harrisonburg, Virginia, USA; f Department of Biological Sciences, University of Pittsburgh, Pittsburgh, Pennsylvania, USA

## Abstract

We report here the genome sequences of three newly isolated phages that infect *Mycobacterium smegmatis* mc^2^155. Phages Findley, Hurricane, and TBond007 were discovered in geographically distinct locations and are related to cluster K mycobacteriophages, with Findley being similar to subcluster K2 phages and Hurricane and TBond007 being similar to subcluster K3 phages.

## GENOME ANNOUNCEMENT

A large collection of completely sequenced mycobacteriophages reveals their considerable genetic diversity, and the mycobacteriophages can be grouped into more than 30 genomic types based on overall nucleotide sequence similarity (1). One of these groups, cluster K, contains temperate phages with broad host ranges that infect both *Mycobacterium smegmatis* and *Mycobacterium tuberculosis* (2).

Phages Findley, Hurricane, and TBond007 were isolated from geographically dispersed locations in New York, Washington, and Texas, respectively, using *M. smegmatis* mc^2^155 as the host. Electron microscopy shows that all three phages are morphologically members of the family *Siphoviridae*. After isolation and purification, double-stranded DNA was sequenced using Illumina MiSeq, and single-end reads of 140 bp were assembled using Newbler to yield a major contig for each with at least 1,000-fold coverage, except for the contig of Hurricane, which has 54-fold coverage. The genomes of Findley, Hurricane, and TBond007 are 58,150 bp, 61,318 bp, and 61,145 bp long, respectively, and coverage analysis shows they possess *cos* ends with 10- to 11-nucleotide single-stranded 3′ extensions. All three phages have a G+C content of 67%, which is similar to that of *M. smegmatis*.

Protein-coding genes were identified using Glimmer (3) and GeneMark (4), followed by manual inspection and revision using DNA Master (http://cobamide2.bio.pitt.edu/computer.htm). From a comparative analysis using previously described parameters (5), Findley was assigned to cluster K2, and both Hurricane and TBond007 were assigned to cluster K3 (2, 6, 7). Ninety-four to 100 protein-coding genes are predicted for each phage, accounting for 92.5 to 94.4% of the genome-coding capacities. Protein-coding genes with assigned functions constitute 36 to 51% of the total genomes. No tRNA genes were identified in any of the three phages. BLASTN analysis shows that Hurricane is closely related to TBond007, sharing 95% nucleotide identity, whereas Findley only shares 75% nucleotide identity with the other two phages; the strongest regions of similarity are in the genome left arms containing the virion structure and assembly genes. All genes are transcribed in the forward direction, except for three or four genes at the center of each genome. Like other cluster K phages, all three phages contain multiple copies of 13-bp start-associated sequences (13, 16, and 17 copies in Findley, Hurricane, and TBond007, respectively), with no more than one mismatch to the consensus 5′-GGGATAGGAGCCC sequence (2). They also contain six to nine copies of the extended start-associated sequences upstream of subsets of these, as in other cluster K phages (2).

All three phages have tyrosine integrase and immunity repressor genes, indicating temperate lifestyles, as with other cluster K phages (2). Phage attachment *attP* sites were identified in Hurricane and TBond007, sharing common cores with an *attB* site overlapping the *M. smegmatis* mc^2^155 tRNA-Lys gene (Msmeg_4746), similar to other K3 phages (2); the Findley *attP* site corresponds to an *attB* site in the transfer-messenger RNA (tmRNA) gene (2) and was confirmed experimentally. The Hurricane immunity repressor (gene *46*; 150 amino acids) has 83.5 to 85% overall amino acid sequence identity with the repressors of other K3 phages (including TBond007), with near identity over the N-terminal 105 residues but highly divergent C termini. Further experiments are needed to determine if K1 and K3 phages share immune specificity.

### Accession number(s).

The GenBank accession numbers for Findley, Hurricane, and TBond007 are MF140411, MF373841, and KX683428, respectively.
